# *NUDT2* Disruption Elevates Diadenosine Tetraphosphate (Ap_4_A) and Down-Regulates Immune Response and Cancer Promotion Genes

**DOI:** 10.1371/journal.pone.0154674

**Published:** 2016-05-04

**Authors:** Andrew S. Marriott, Olga Vasieva, Yongxiang Fang, Nikki A. Copeland, Alexander G. McLennan, Nigel J. Jones

**Affiliations:** 1 Department of Biochemistry, Institute of Integrative Biology, University of Liverpool, Liverpool, Merseyside, United Kingdom; 2 Department of Functional and Comparative Genomics, Institute of Integrative Biology, University of Liverpool, Liverpool, Merseyside, United Kingdom; 3 Division of Biomedical and Life Sciences, University of Lancaster, Lancaster, Lancashire, United Kingdom; University of Jaén, SPAIN

## Abstract

Regulation of gene expression is one of several roles proposed for the stress-induced nucleotide diadenosine tetraphosphate (Ap_4_A). We have examined this directly by a comparative RNA-Seq analysis of KBM-7 chronic myelogenous leukemia cells and KBM-7 cells in which the *NUDT2* Ap_4_A hydrolase gene had been disrupted (NuKO cells), causing a 175-fold increase in intracellular Ap_4_A. 6,288 differentially expressed genes were identified with *P* < 0.05. Of these, 980 were up-regulated and 705 down-regulated in NuKO cells with a fold-change ≥ 2. Ingenuity^®^ Pathway Analysis (IPA^®^) was used to assign these genes to known canonical pathways and functional networks. Pathways associated with interferon responses, pattern recognition receptors and inflammation scored highly in the down-regulated set of genes while functions associated with MHC class II antigens were prominent among the up-regulated genes, which otherwise showed little organization into major functional gene sets. Tryptophan catabolism was also strongly down-regulated as were numerous genes known to be involved in tumor promotion in other systems, with roles in the epithelial-mesenchymal transition, proliferation, invasion and metastasis. Conversely, some pro-apoptotic genes were up-regulated. Major upstream factors predicted by IPA^®^ for gene down-regulation included NFκB, STAT1/2, IRF3/4 and SP1 but no major factors controlling gene up-regulation were identified. Potential mechanisms for gene regulation mediated by Ap_4_A and/or *NUDT2* disruption include binding of Ap_4_A to the HINT1 co-repressor, autocrine activation of purinoceptors by Ap_4_A, chromatin remodeling, effects of NUDT2 loss on transcript stability, and inhibition of ATP-dependent regulatory factors such as protein kinases by Ap_4_A. Existing evidence favors the last of these as the most probable mechanism. Regardless, our results suggest that the NUDT2 protein could be a novel cancer chemotherapeutic target, with its inhibition potentially exerting strong anti-tumor effects via multiple pathways involving metastasis, invasion, immunosuppression and apoptosis.

## Introduction

Nudix hydrolases regulate the levels of a wide variety of canonical and modified nucleotides and some non-nucleotide phosphorylated substrates as well as participating in essential processes such as mRNA decapping [1, 2]. One of the best studied is mammalian NUDT2. This enzyme has been isolated from many sources [3, 4] and its principal substrate is believed to be diadenosine 5′,5′′′-*P*^1^,*P*^4^-tetraphosphate (Ap_4_A). In animal cells, Ap_4_A can be synthesized by most aminoacyl-tRNA synthetases, DNA ligases, firefly luciferase and acyl-CoA synthetases while a further range of enzymes is able to do so in plants, fungi and bacteria [5–7]. Synthesis usually involves transfer of AMP from an acyl-AMP or enzyme-AMP reaction intermediate to an ATP acceptor. It can also be degraded by a number of enzymes in addition to NUDT2, including FHIT [8], aprataxin [9] and non-specific phosphodiesterases [3]. However, NUDT2 is believed to be principally responsible for maintaining the low level of intracellular Ap_4_A [10–12].

An increase in Ap_4_A resulting from activation of synthesis, inhibition of degradation or both has been implicated in several intracellular processes. Genotoxic, thermal and other stresses lead to increased Ap_4_A [13–17] and so Ap_4_A has been implicated in the regulation of DNA replication after DNA damage and in promoting apoptosis [17–19]. Ap_4_A may also be raised in response to external ligands and act as an intracellular second messenger [20–22]. It also acts as an extracellular messenger through its interaction with a number of P2-type receptors [23]. Ap_4_A is also a ligand for a number of proteins including a multiprotein complex containing DNA polymerase-α [24, 25], protein kinases [26–28], uracil-DNA glycosylase [29], protein chaperones [30], the HINT1 tumor suppressor [31], 5′-nucleotidase II [32], CBS domain proteins [33, 34] and CFIm25 [35], but in most cases the significance of this binding is not clear. Of particular interest, however, is the possibility that Ap_4_A may act as a transcriptional regulator. It has been suggested that an increased level of Ap_4_A induced in mast cells by external factors activates the expression of a subset of genes controlled by the MITF and USF2 transcription factors by binding to and displacing the inhibitory HINT1 protein from these factors [10, 31, 36].

In order to determine whether transcriptional regulation by Ap_4_A is confined to relatively few genes or is more widespread, we have analysed the transcriptome of a knockout derivative of the KBM-7 chronic myeloid leukaemia (CML) cell line [37] in which the intracellular level of Ap_4_A has been increased 175-fold by disruption of the *NUDT2* gene (KBM-7-NuKO, referred to hereafter as NuKO). These cells show profound changes in gene expression compared to the parent KBM-7 cell line with a total of 6288 significantly differentially expressed genes (DEGs) identified. Ingenuity^®^ Pathway Analysis was used to highlight the gene networks and metabolic and signaling pathways affected, revealing down-regulation of interferon, inflammatory and innate immune responses and up-regulation of processes involving MHC class II antigens. In addition, many of the most strongly affected genes have roles in promoting cancer metastasis and invasion, suggesting that NUDT2 may offer a novel, pleiotropic target for cancer chemotherapy.

## Materials and Methods

### Cells

The KBM-7 reference clone B (product no. P00174E07) and the KBM-7-NuKO derivative (P01289H04) in which the *NUDT2* gene has been inactivated by retroviral gene-trap insertion [38] were obtained from Haplogen and maintained at 37°C in 5% (v/v) CO_2_/air in Isocoves modified Eagle medium (IMEM, Sigma) supplemented with 10% (v/v) Foetal Bovine Serum (Sigma), 2 mM L-glutamine (Sigma) and 100 μg mL ^-1^ penicillin-streptomycin (Sigma).

### Measurement of Ap_4_A and derivatives

The level of intracellular Ap_4_A in log phase KBM-7 and NuKO cells was determined as previously described using a sensitive luminometric assay with slight modifications for use with suspension cells [17, 39]. Cells were harvested from suspension by centrifugation at 500 *g* for 5 min and used for nucleotide extraction. Ap_4_A was also measured in the growth medium supernatant from these cells, which was filtered through a 0.2 μm Millipore filter, deproteinized with 10% TCA, then assayed as above. ADP-ribosylated derivatives of Ap_4_A (ADPR-Ap_4_A) were separated by ion-exchange chromatography and identified and assayed as previously described [17].

### Growth inhibition assays

Cells (2 x 10^5^) were seeded into 25 cm^2^ flasks containing 7 mL of growth medium. Chemical agents were added as stated and cells grown for 96 h at 37°C after which cultures were centrifuged at 500 *g* for 5 min, cells resuspended in fresh medium, and counted using a haemocytometer. Average counts were normalized to the cell count of the untreated culture.

### RNA-Seq analysis: cDNA library preparation and sequencing

Three independent samples of total RNA were prepared from both KBM-7 and NuKO cells. RNA extraction was performed using a Qiagen RNeasy mini kit with QIAshredder, and the quantity and quality determined using a Nanodrop and Agilent Bioanalyzer. For each of the six samples, 10 μg of RNA was DNase-treated using an Ambion TURBO DNA-*free*™ kit and subsequently purified using AMPure XP beads. 2 μg of the DNase-treated total RNA was then subjected to rRNA depletion using the Ribo-Zero Gold (Human/Mouse/Rat) kit and purified again with Ampure XP beads. Successful depletion was assessed using a Qubit fluorometer and Agilent 2100 Bioanalyzer and all of the depleted RNA was used for the RNA-Seq library preparation using the ScriptSeq v2 protocol. Following 15 cycles of amplification the libraries were purified using Ampure XP beads. Each library was quantified using Qubit and the size distribution assessed using the Agilent 2100 Bioanalyzer. The final libraries were pooled in equimolar amounts using the Qubit and Bioanalyzer data. The quantity and quality of each pool was assessed with the Bioanalyzer and by qPCR using the KAPA Library Quantification kit for Illumina platforms on a Roche LC480II Light Cycler according to manufacturer's instructions. The template DNA was denatured according to the protocol described in the Illumina cBot user guide and loaded at a concentration of 9 pM. Sequencing was carried out on one lane of an Illumina HiSeq 2000 with version 3 chemistry generating 2 × 100 bp paired end reads. Quality control was maintained with a 1% PhiX spike-in.

### Bioinformatic analysis of RNA-Seq data

Basecalling and de-multiplexing of indexed reads for each sample library was performed using CASAVA 1.8.2 (Illumina). Raw fastq files were processed using Cutadapt 1.2.1 [40] with option “-O 3” set to remove adapter sequences of 3 bp or more. Reads were further trimmed using Sickle 1.200 to remove low quality bases and finally reads <10 bp were removed. The TopHat2 aligner version 2.0.10 [41] was used to align the trimmed R1-R2 read pairs to the human reference genome assembly GRCh38, which contains 64,253 genes. Default parameters were used except for the library type option, which was set to “fr-secondstrand” for all samples as the kit used produced a second-strand library type (R1 is expected to map on the 5′→3′ strand and R2 on the 3′→5′ strand). Reads aligning to the reference in more than one position were discarded and FKPM values (fragments per kilobase transcript per million reads mapped) calculated. Differential gene expression analysis was conducted in the R environment using the edgeR package [42]. The count data were normalised across libraries using the Trimmed Mean M-values (TMM) method in edgeR with default parameters. Tagwise dispersion parameters were estimated and then used for log_2_FC (log_2_ Fold Change) estimation and testing in edgeR using the Likelihood Ratio (LR test) [43]. *P* values associated with log_2_FC were adjusted for multiple testing using the False Discovery Rate (FDR) approach [44]. Significant DEGs were defined as those with an FDR-adjusted *P* value < 0.05. All original RNA-Seq data produced in this study have been submitted to the EMBL-EBI ArrayExpress database under accession number E-MTAB-4104.

### RT-PCR analysis of selected genes

RNA extraction was performed using a Qiagen RNeasy mini kit with QIAshredder and cDNA was synthesized using a Bioline Tetro cDNA synthesis kit, both according to the manufacturer’s instructions. The cDNA was then quantitated by PCR using Maxima SYBR Green master mix (Thermo) and a StepOnePlus™ Real Time PCR system (Applied Biosystems). Primers were obtained from Sigma and are listed in S1 Table. The 2^-ΔΔCt^ method was used to determine relative transcript levels using the housekeeping GAPDH gene to normalize the data [45].

### Pathway analysis

Genes showing ≥ 2-fold up- or down-regulation with an FDR-adjusted *P* value < 0.05 were analyzed through the use of QIAGEN Ingenuity^®^ Pathway Analysis software (IPA^®^, QIAGEN, Redwood City, http://www.ingenuity.com) in order to assign them to different functional networks. IPA^®^ uses the manually curated Ingenuity® Knowledge Base, which contains information from several gene and protein expression, interaction and annotation databases such as IntACT, BIND and MiPs, as well as from the published literature [46]. We also used IPA to identify functionally related genes that correspond to specific canonical pathways that were most significant to the data set from a collection of 200 curated metabolic, cell-signaling cascade and disease-associated pathways. Fisher’s exact test of independence was used to calculate the probability that the association between the genes in the dataset and the canonical pathway can be explained by chance alone. Finally, we used the IPA upstream regulator analysis to identify factors that may control the genes and pathways highlighted by network analysis to provide testable hypotheses for gene regulation by Ap_4_A.

## Results

### Level of Ap_4_A in KBM-7-NuKO cells

**Fig 1:**
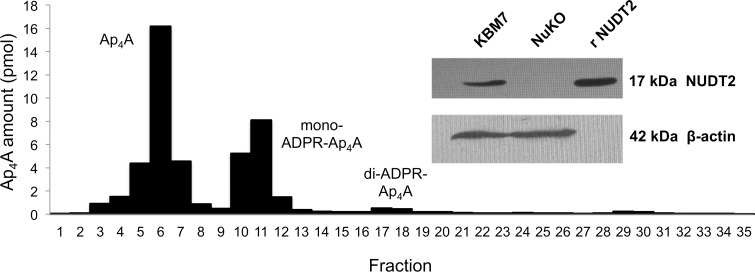
Ion-exchange chromatography of Ap_4_A extracted from KBM-7-NuKO cells and (inset) western blot analysis of cell extracts for the presence of NUDT2 protein. A nucleotide extract from NuKO cells was subjected to ion-exchange chromatography and fractions assayed luminometrically for Ap_4_A as described in Materials and Methods. Inset: a sample of recombinant NUDT2 and protein extracts of KBM-7 and KBM-7 NuKO cells were subjected to polyacrylamide gel electrophoresis and subsequent nitrocellulose blots probed for the presence of NUDT2 with rabbit polyclonal anti-NUDT2 (Santa Cruz) followed by detection with HRP-conjugated goat-anti-rabbit IgG and ECL visualization (ECL Select, GE Healthcare). Mouse β-actin was detected with HRP-conjugated goat-anti-mouse IgG (Santa Cruz).

The parent KBM-7 line used in this study contains the *BCR-ABL1* gene fusion and potentially inactivating mutations in *TP53* and *NOTCH1*, but lacks the other common genetic aberrations found in myeloid malignancies [38]. It expresses the majority of annotated proteins from a wide range of signaling pathways, making it a suitable cell line for this study. The complete absence of NUDT2 protein from the NuKO *NUDT2* disruptant was confirmed by Western blotting (Fig 1). The steady-state concentration of intracellular Ap_4_A in unstressed mammalian cells is typically in the range 0.1–1.0 pmol/10^6^ cells (0.05–0.5 μM), the exact amount being species- and cell type-dependent [17, 47]. Log phase KBM-7 cells had a level of 0.21±0.02 (*n* = 3) pmol/10^6^ cells. However, the NuKO derivative had a 175-fold increased level of 36.9±0.3 (*n* = 3) pmol/10^6^ cells, providing the clearest evidence yet that Ap_4_A is an important NUDT2 substrate *in vivo* and that this enzyme plays an essential role in maintaining the low background level of Ap_4_A. Note that an Ap_4_A content of 1 pmol/10^6^ cells equates roughly to an intracellular concentration of 0.5 μM if uniformly distributed [17] so the level in NuKO cells will be around 20 μM. Regarding whether this high level and the resulting changes in the cells reported here are biologically relevant, we have previously measured up to 20 μM Ap_4_A in DNA repair-defective cells treated with mitomycin C [17] while a concentration as high as 775 μM has been reported in FCεR1-activated mast cells [31]. Chromatographic analysis of the Ap_4_A from NuKO cells showed that about 35% was present in the form of ADP-ribosylated derivatives (ADPR-Ap_4_A), mainly mono-ADPR-Ap_4_A (Fig 1). We have previously shown that ADP-ribosylation of Ap_4_A by PARP1 and PARP2 in Chinese hamster EM9 cells and mouse embryo fibroblasts occurs in response to DNA damage [17]; however, it appears that the high level of Ap_4_A here is subject to constitutive ADP-ribosylation.

### RNA-Seq and differential gene expression analysis

Ap_4_A has been reported to activate the transcription of subsets of genes controlled by the transcription factors MITF and USF2 [31, 36]. In view of this, and to further explore the phenotype of the *NUDT2* knockout cells, we carried out a comparative analysis of the transcriptomes of KBM-7 and NuKO cells by RNA-Seq to identify DEGs. An average of 46.1 million pairs of 100 bp paired-end reads per sample were generated that aligned to the reference human genome. Alignment results are summarized in Table 1, showing the number and percentage of reads mapped for each sample. Mapping percentages for the six samples were between 80.2 and 81.3%. 31,177 (48.5%) of the 64,253 reference genes had at least one read aligned while 33,076 genes had no read aligned from any of the six samples.

**Table 1:** Number and percentage of reads mapped to the human reference genome.

Sample	Reads to align^a^	Reads aligned to genome	% of alignment	Reads aligned in pairs	% reads aligned in pairs	% concordant pairs^b^
KBM7.1	52,648,492	46,517,676	88.4	42,437,310	80.6	70.7
KBM7.2	48,836,484	43,222,156	88.5	39,472,258	80.8	70.5
KBM7.3	53,927,610	47,635,707	88.3	43,402,072	80.5	70.8
N2KO.1	63,959,114	56,363,684	88.1	51,618,308	80.7	71.6
N2KO.2	64,109,906	56,893,008	88.7	52,135,004	81.3	72.5
N2KO.3	59,263,006	52,123,251	88.0	47,532,894	80.2	70.5

^a^Sum of R1 and R2 reads used in the alignment

^b^Percentage of read pairs both of whose reads aligned to the same chromosome.

All percentages were calculated based on the total number of reads to align.

**Fig 2:**
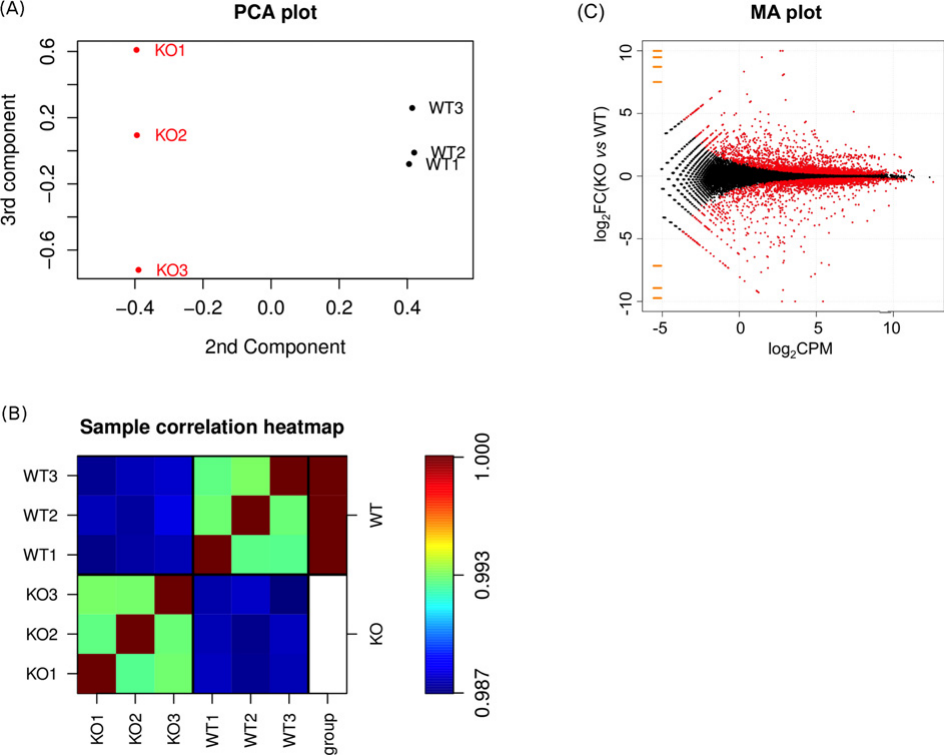
Principal component analysis (PCA), correlation analysis and MA plot of differential gene expression data. (A) PCA plot of log2 gene expression data showing the 2^nd^ and 3^rd^ principal components. (B) Heatmap visualization of the Pearson correlation coefficients of log_2_ gene expression between samples. The three samples from wild type KBM-7 cells are labeled WT1-WT3 and those from the KBM-7-NuKO cells KO1-KO3. (C) MA plot showing the distribution of mean gene expression levels (log_2_Counts Per Million mapped reads) against log_2_ Fold-Change (KO *vs* WT) for individual gene responses. Low expression genes (log_2_CPM < −5) are colored orange. Significant differentially expressed genes (DEGs) are colored red; genes showing no change in expression are colored black.

The difference in gene expression profiles between the two cell types is illustrated in the Principal Component Analysis (PCA) plot of log2 gene expression data shown in Fig 2A. The triplicate samples of each cell type are grouped well away from each other, indicating a high degree of differential gene expression between them. Furthermore, the heatmap of the Pearson correlation coefficients in Fig 2B indicated that the expression profiles for the three samples from the same cell type were much more closely correlated than samples from different cell types, showing that the effect of *NUDT2* knockout on gene expression was much stronger than the influence of any technical or biological variations between samples. The heatmap also shows a very high correlation (R >0.99) among samples from the same cell type. Thus, we can conclude that the differential gene expression detected here is statistically very robust.

Of the 31,177 reads mapped (S2 Table) a total of 6,288 DEGs were identified with a *P*-value (FDR-adjusted) < 0.05, of which 2,550 were up-regulated and 2,285 down-regulated with a fold-change ≥ 1.2 (Fig 2C and S3 Table). The MA plot in Fig 2C shows a fairly symmetrical distribution of up- and down-regulated genes at all levels of expression. Of these genes, 980 were up-regulated and 705 down-regulated with a fold-change ≥ 2. In both cases, 88% had FPKM ≥ 0.3 for one or both of the WT and KO datasets. The 40 most strongly down- and up-regulated annotated genes are shown in Tables 2 and 3 respectively. Note that many of these genes had zero read counts for either the WT or KO datasets necessitating the addition of a small zero-offset pseudocount by the edgeR software in order to calculate log_2_FC [42].

**Table 2:** The 40 most strongly down-regulated genes in KBM7-NuKO cells.

Gene id	Approved gene name	Fold change	FDR-*P*, KO vs WT^1^	FPKM WT^2^	FPKM KO^2^
GFRA1	GDNF family receptor alpha 1	3221.0	4.38E-298	2.548	0.000
ZNF660	zinc finger protein 660	1785.1	5.30E-174	2.094	0.000
DZIP1	DAZ interacting zinc finger protein 1	1364.6	0.00E+00	10.206	0.007
CROT	carnitine O-octanoyltransferase	623.2	2.81E-60	0.958	0.000
EDIL3	EGF-like repeats and discoidin I-like domains 3	590.1	3.02E-68	0.785	0.000
PHACTR3	phosphatase and actin regulator 3	566.5	9.06E-65	0.711	0.000
NUDT2	nudix (NDP- linked moiety X)-type motif 2	545.0	1.03E-181	13.295	0.018
RAB42	RAB42, member RAS oncogene family	267.5	3.31E-31	0.936	0.000
MPZL2	myelin protein zero-like 2	260.3	0.00E+00	10.580	0.039
IFI44L	interferon-induced protein 44-like	224.3	0.00E+00	5.862	0.026
GALM	galactose mutarotase (aldose 1-epimerase)	203.4	1.84E-77	1.908	0.006
CRISP3	cysteine-rich secretory protein 3	161.1	9.10E-96	3.206	0.017
TP53TG1	TP53 target 1 (non-protein coding)	160.9	7.09E-19	1.194	0.000
MGMT	O-6-methylguanine-DNA methyltransferase	157.9	1.56E-18	0.337	0.000
LUM	lumican	142.9	9.87E-15	0.360	0.000
misc_RNA	Y-RNA-like misc_RNA, chromosome 8	137.3	1.76E-15	9.261	0.000
BRINP3	bone morphogenetic/retinoic acid inducible protein 3	135.8	0.00E+00	32.885	0.243
NKX2-2	NK2 homeobox 2	125.3	8.96E-15	0.457	0.000
IGF2BP1	insulin-like growth factor 2 mRNA binding protein 1	121.7	0.00E+00	8.783	0.071
GJA1	gap junction protein, alpha 1, 43kDa	120.4	3.14E-174	4.721	0.037
ABCC6	ATP-binding cassette, sub-family C, member 6	110.5	4.57E-13	0.111	0.000
GYPE	glycophorin E (MNS blood group)	110.5	2.42E-12	0.418	0.000
ADGRL3	adhesion G protein-coupled receptor L3	109.0	6.96E-33	0.203	0.001
RAG2	recombination activating gene 2	107.5	4.59E-160	4.217	0.037
FIGN	fidgetin	87.7	1.97E-38	0.394	0.004
CPED1	cadherin-like and PC-esterase domain containing 1	87.1	4.13E-63	0.925	0.009
TMEM254	transmembrane protein 254	84.4	9.42E-90	2.303	0.025
GLDC	glycine dehydrogenase (decarboxylating)	83.9	5.13E-10	0.136	0.000
JCHAIN	joining chain of multimeric IgA and IgM	74.6	4.58E-27	1.160	0.011
CNTNAP5	contactin associated protein-like 5	72.1	3.90E-08	0.101	0.000
TM4SF1	transmembrane 4 L six family member 1	70.3	4.74E-24	0.502	0.005
CXCL10	chemokine (C-X-C motif) ligand 10	66.1	1.78E-181	30.704	0.463
KYNU	kynureninase	64.5	1.46E-162	0.845	0.013
EVA1A	eva-1 homolog A (C. elegans)	57.3	4.61E-30	0.929	0.014
CNTNAP4	contactin associated protein-like 4	54.8	2.13E-33	0.378	0.006
HPGD	hydroxyprostaglandin dehydrogenase 15-(NAD)	52.1	5.40E-19	0.246	0.003
TSPAN7	tetraspanin 7	51.4	7.66E-06	0.125	0.000
CD200	CD200 molecule	51.1	1.26E-98	2.669	0.051
OAS2	2'-5'-oligoadenylate synthetase 2, 69/71kDa	49.4	4.24E-278	5.029	0.100
CXCL11	chemokine (C-X-C motif) ligand 11	47.1	4.54E-120	6.138	0.128

^1^False discovery rate-adjusted *P*-value

^2^Fragments per kilobase of transcript per million mapped reads, a measure of transcript abundance in KBM7 wild type (WT) and KBM7-NuKO (KO) knockout cells

**Table 3:** The 40 most strongly up-regulated genes in KBM7-NuKO cells.

Gene id	Approved gene name	Fold change	FDR-*P*, KO vs WT^1^	FPKM WT^2^	FPKM KO^2^
LINC01224	long intergenic non-protein coding RNA 1224	1832.2	1.56E-149	0.000	10.839
ZNF483	zinc finger protein 483	1649.2	9.98E-143	0.000	2.201
OVOL1	ovo-like zinc finger 1	718.9	5.18E-50	0.000	1.434
FAM162B	family with sequence similarity 162, member B	323.3	3.96E-30	0.000	2.382
NAP1L2	nucleosome assembly protein 1-like 2	280.2	9.86E-180	0.018	5.828
ZNF544	zinc finger protein 544	264.4	1.02E-171	0.007	1.991
SLC1A1	solute carrier family 1	110.2	1.06E-11	0.000	0.211
TACC2	transforming, acidic coiled-coil containing protein 2	107.6	8.60E-11	0.000	0.043
ATP10B	ATPase, class V, type 10B	76.2	3.91E-08	0.000	0.051
EMC10	ER membrane protein complex subunit 10	67.6	1.74E-172	0.021	1.475
SGPP2	sphingosine-1-phosphate phosphatase 2	62.5	2.32E-41	0.013	0.992
GALNT5	polypeptide N-acetylgalactosaminyltransferase 5	59.8	1.73E-25	0.006	0.465
NHSL2	NHS-like 2	51.9	9.76E-06	0.000	0.098
ECT2L	epithelial cell transforming 2 like	49.5	1.28E-05	0.000	0.080
SERPINB10	serpin peptidase inhibitor, clade B, member 10	47.1	2.28E-05	0.000	0.131
HLA-DOA	major histocompatibility complex, class II, DOA	47.0	9.78E-05	0.000	0.088
FAM155A	family with sequence similarity 155, member A	42.5	0.000444026	0.000	0.038
PAK3	p21 protein (Cdc42/Rac)-activated kinase 3	42.2	7.28E-05	0.000	0.030
DNMT3L	DNA (cytosine-5-)-methyltransferase 3-like	39.8	0.000129366	0.000	0.159
THY1	Thy-1 cell surface antigen	39.8	0.000129366	0.000	0.050
ABCA8	ATP-binding cassette, sub-family A (ABC1), 8	37.8	0.002009234	0.000	0.025
CYP26B1	cytochrome P450, family 26B, polypeptide 1	37.4	0.000229216	0.000	0.055
ANXA3	annexin A3	35.3	0.002276797	0.000	0.052
BASP1	brain abundant, membrane attached signal protein 1	35.2	0	4.294	151.21
MIXL1	Mix paired-like homeobox	35.0	0.000407311	0.000	0.129
SDC4	syndecan 4	34.8	1.39E-55	0.053	1.918
CELA1	chymotrypsin-like elastase family, member 1	32.9	0.009226334	0.000	0.259
TMEM176B	transmembrane protein 176B	32.8	3.69E-09	0.011	0.502
SNX19	sorting nexin 19	32.8	0.011374951	0.000	0.030
NAV3	neuron navigator 3	32.6	0.00158859	0.000	0.022
LINC01124	long intergenic non-protein coding RNA 1124	32.6	0.001588576	0.000	0.115
HS6ST3	heparan sulfate 6-O-sulfotransferase 3	32.5	0.000722541	0.000	0.031
PAK6	p21 protein (Cdc42/Rac)-activated kinase 6	32.5	0.000722541	0.000	0.037
LINC00925	long intergenic non-protein coding RNA 925	32.0	8.37E-20	0.006	0.233
AOC1	amine oxidase, copper containing 1	30.6	2.14E-58	0.059	1.881
C1orf204	chromosome 1 open reading frame 204	30.2	0.001942513	0.000	0.091
NDRG4	NDRG family member 4	30.1	0.00128289	0.000	0.023
NHS	Nance-Horan syndrome	30.1	0.001597534	0.000	0.022
NIM1K	NIM1 serine/threonine protein kinase	29.3	6.57E-17	0.012	0.429
A4GALT	alpha 1,4-galactosyltransferase	29.1	3.48E-08	0.008	0.301

^1^False discovery rate-adjusted *P*-value

^2^Fragments per kilobase of transcript per million mapped reads, a measure of transcript abundance in KBM7 wild type (WT) and KBM7-NuKO (KO) knockout cells

**Fig 3:**
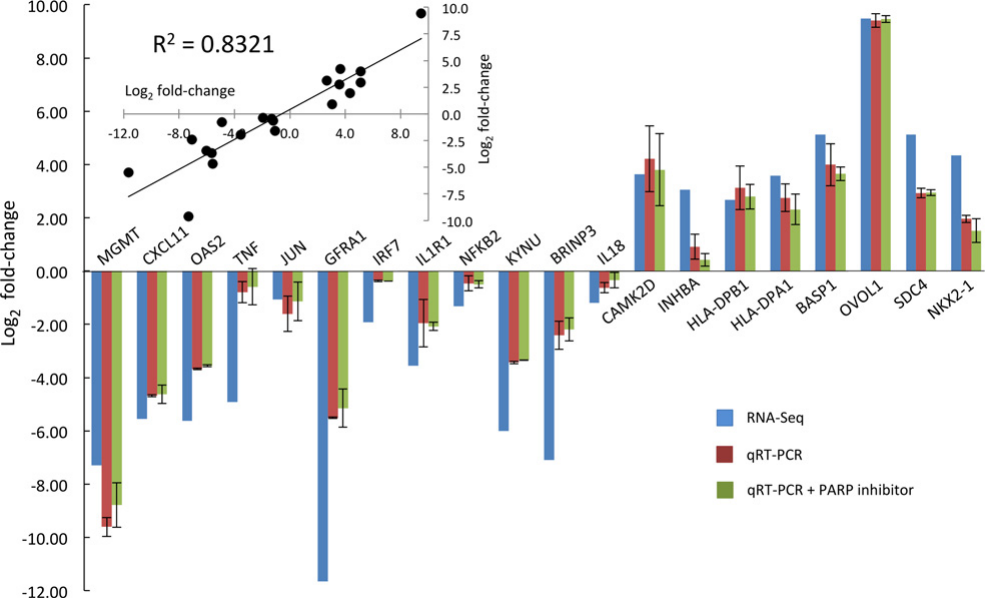
Validation of RNA-Seq data for differential gene expression by qRT-PCR. qRT-PCR analysis was performed on selected RNAs from KBM-7 and NuKO cells in the presence and absence of 100 nM of the PARP inhibitor KU-0058948 **using** the primers listed in S1 Table as described in Materials and Methods and the log_2_ fold-change in expression plotted beside those obtained by RNA-Seq analysis. Inset: simple correlation plot of the log_2_ fold-changes in expression obtained by RNA-Seq (*x*-axis) and qRT-PCR without PARP inhibitor (*y*-axis).

The RNA-Seq analysis was validated by performing real-time qRT-PCR on a selection of genes representing various affected pathways (Fig 3). These results confirmed the direction of regulation (up or down) for all genes studied. The magnitude of change was also similar for the majority of genes, with a correlation coefficient of 0.83 between the two data sets (Fig 3, inset). However, for some genes with a zero value of FPKM for one of the samples in the RNA-Seq analysis, the use of the pseudocount method by edgeR to calculate a fold-change has led to a significantly different value, e.g. *GFRA1* and *TNF*. Nevertheless, the values calculated by edgeR are used in the following discussions as they are available for all genes and are still a good relative indication of the change in expression. In order to show that the observed differential gene expression correlates solely with increased Ap_4_A rather than the related ADPR-Ap_4_A derivatives, qRT-PCR analysis was also performed with RNA extracted from NuKO cells grown in the presence of 100 nM KU-0058948, a PARP1 and PARP2 inhibitor that prevents the synthesis of ADPR-Ap_4_A species [17]. The results were very similar to those obtained in the absence of KU-0058948 (Fig 3), showing that, for these genes at least, ADPR-Ap_4_A is not the cause of the differential expression. The function of ADPR-Ap_4_A, if any, is still unclear.

### Ingenuity^®^ Pathway Analysis

In order to place the gene expression data into a biological context, Ingenuity^®^ Pathway Analysis (IPA^®^) software was used to assign the DEGs to known canonical pathways and functional networks in order to predict the biological functions of the transcriptional changes. For simplicity, the initial analysis included only genes that were up- or down-regulated by ≥ 2-fold (*P* < 0.05); however, where present in the resulting pathways and networks, genes up- or down-regulated by ≥ 1.2 were also considered to be of potential interest as there is no biological justification for a cut-off value of 2. It was found that the DEGs mapped to a large number of pathways with a significant enrichment score (–log(*P*-value)) (S4 Table). Top ranked within both up- and down-regulated gene sets were signaling pathways related to immunity and inflammation. Pathways associated primarily with the innate immune response, such as activation of interferon regulatory factors (IRFs) by pattern recognition receptors (PRRs), and inflammation were specifically enriched in the down-regulated set of genes while functions associated with MHC class II antigens were specific for the set of up-regulated genes (Table 4). The predominance of these pathways in the dataset may reflect the myeloid nature of the KBM-7 cell line [37]. These pathways are discussed in detail below.

**Table 4:** Major IPA^®^ pathways and functions involving differentially regulated genes.

**Top Down-regulated Canonical Pathways**	***P*-value**
Activation of IRF by cytosolic pattern recognition receptors	4.04E-09
IL-1 signalling	1.80E-07
Recognition of bacteria and viruses by pattern recognition receptors	5.32E-07
Altered T cell and B cell signalling in rheumatoid arthritis	1.29E-06
B cell development	6.06E-06
**Top Upstream Regulators**	**Predicted activation**
IFNL1	Inhibited
IFNA2	Inhibited
IL1RN	Activated
TCR	
IFNG	Inhibited
**Top Diseases and Biofunctions**	***P*-value**
Antimicrobial response	8.33E-11–1.21E-11
Inflammatory response	3.99E-03–1.21E-11
Dermatological diseases and conditions	3.32E-03–1.62E-10
Cancer	4.11E-03–6.54E-09
Infectious disease	3.32E-03–1.41E-08
**Top Up-regulated Canonical Pathways**	***P*-value**
Allograft rejection signaling	1.10E-08
OX40 signaling pathway	5.15E-08
B cell development	4.09E-07
Antigen presentation pathway	5.67E-07
Autoimmune thyroid disease signaling	2.03E-06
**Top Upstream Regulators**	**Predicted activation**
SMC3	Activated
PDLIM2	
EBI3	Activated
MYOC	
NEUROG1	
**Top Diseases and Biofunctions**	***P*-value**
Cancer	3.66E-02–9.72E-08
Gastrointestinal disease	3.66E-02–5.57E-06
Hepatic system disease	3.66E-02–9.11E-06
Endocrine system disorders	3.66E-02–4.62E-05
Metabolic disease	3.66E-02–4.62E-05

#### Interferon response and innate immunity

Interferons are important mediators of the innate immune response, which provides an initial vital defence against invading pathogens (viruses, bacteria, protozoa) following interaction of pathogen components with PRRs in various cellular compartments. They can also inhibit cell proliferation, modulate the adaptive immune response, and be pro- or anti-inflammatory, depending on context [48–51]. Submission of the set of 4,835 DEGs with fold change ≥ 1.2 to the Interferome database (v2.01) [52] revealed a subset of at least 1,038 DEGs known to be regulated by Type I IFNs (IFNα and IFNβ) in other systems. Roughly half of these overlapped with the set of 944 showing known regulation by Type II IFNs (IFNγ). Some (56) also showed Type III (IFNλ) regulation with 15 of these potentially unique to Type III (S5 Table).

**Fig 4:**
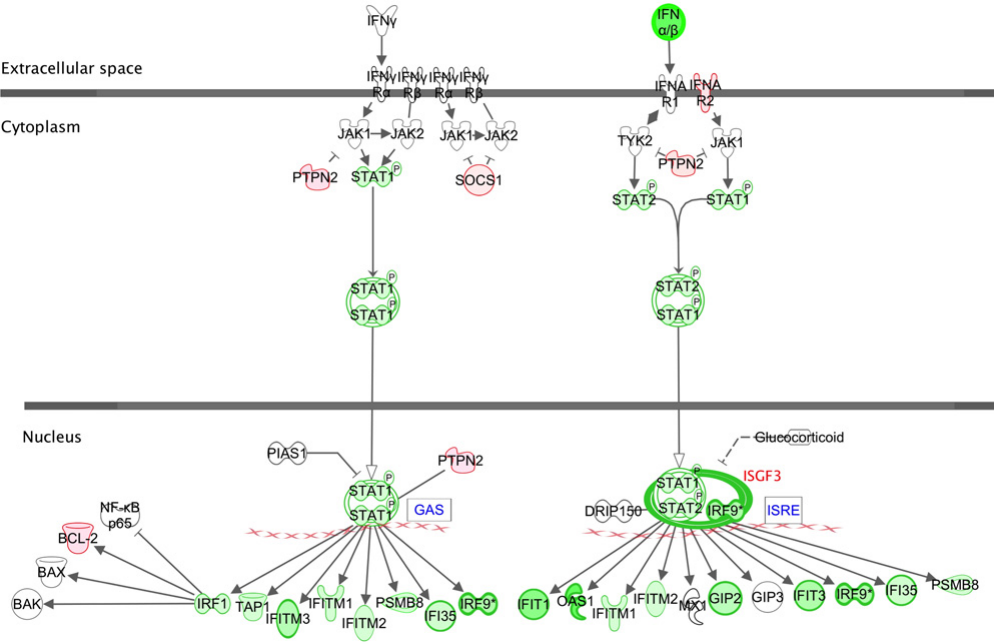
Canonical pathway for Type I and Type II interferon signaling via cell surface IFNR receptors. Down-regulated genes are in green, up-regulated genes in red. Color intensity corresponds to the fold change; bold borders highlight genes with >2-fold change in expression. Lines correspond to physical interactions and arrows to functional relationships between proteins. Solid lines and arrows imply direct relationships and dotted lines and arrows imply indirect relationships. Functional relationships include post-translational modifications, transcription regulation, proteolysis or co-expression. Flat arrowheads indicate inhibition.

Fig 4 shows the IPA^®^ canonical pathway for activation of interferon receptors (IFNRs) by Type I and Type II interferons, with examples of genes found to be differentially expressed in this study highlighted. The majority of functions in this pathway were down-regulated, with expression of *IFNB* being the most strongly affected (15-fold, S3 Table). The JAK-STAT signaling pathways are central to the interferon response. In Type II interferon signaling, activated STAT1 homodimers bind to the GAS (Interferon Gamma Activated Sequence) promoter and induce gene expression while Type I signaling involves the combination of STAT1-STAT2 heterodimers with IRF9 (Interferon Response Factor 9) forming ISGF3 (Interferon Stimulated Gene Factor), which then binds to the ISRE (Interferon-Stimulated Response Element) promoter. *STAT1*, *STAT2* and *IRF9* were all down-regulated (1.4-, 1.8- and 3.7-fold respectively) while the pathway suppressors *SOCS1* and *PTPN2* were up-regulated (1.2–1.4-fold). Though individually slight, the combined effect of these changes could nevertheless be significant. The increases in *SOCS1* and *PTPN2* also show that there is not just a general suppression of gene expression but that negative feedback via these genes is preserved. Finally, several of the STAT-controlled genes that are down-regulated are themselves activators of further IFN response genes, e.g. *IRF1*, *IRF7* and *IRF9* (1.2-, 3.8- and 3.7-fold respectively).

**Fig 5:**
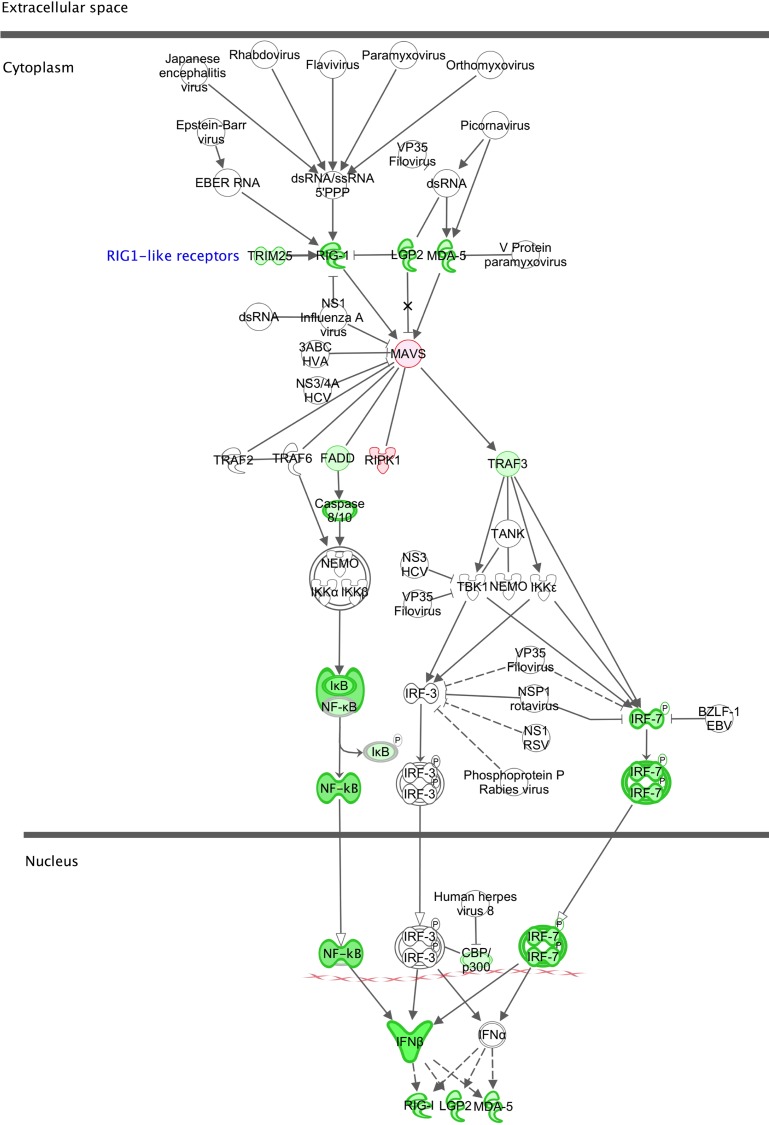
Canonical pathway for the role of RIG-1-like receptors in antiviral innate immunity. Explanation of symbols as in Fig 4.

The canonical pathway in Fig 5 highlights the roles of the three RIG-1-like helicase PRRs of the innate immune response, RIG-1 (DDX58), MDA5 (IFIH1) and LGP2 (DHX58) in the activation of *IFNB* following stimulation by viral double-stranded RNAs and the feedback provided by IFNβ on the expression of these PRRs. All three receptor genes are down-regulated (3.1-, 2.3- and 3.0-fold respectively) (S3 Table) in NuKO cells. In addition, *IFITM2* and *IFITM3*, whose products restrict the entry of many viruses [53], and all four antiviral IFIT family members that bind viral components (*IFIT1*, *2*, *3* and *5*) [54] are down-regulated between 1.6- and 6.8-fold. Other down-regulated anti-viral genes include *PKR*, *GBP1* and *TLR10* [55–58] (S3 Table).

#### Cytokine signaling, inflammation and NF-κB

**Fig 6:**
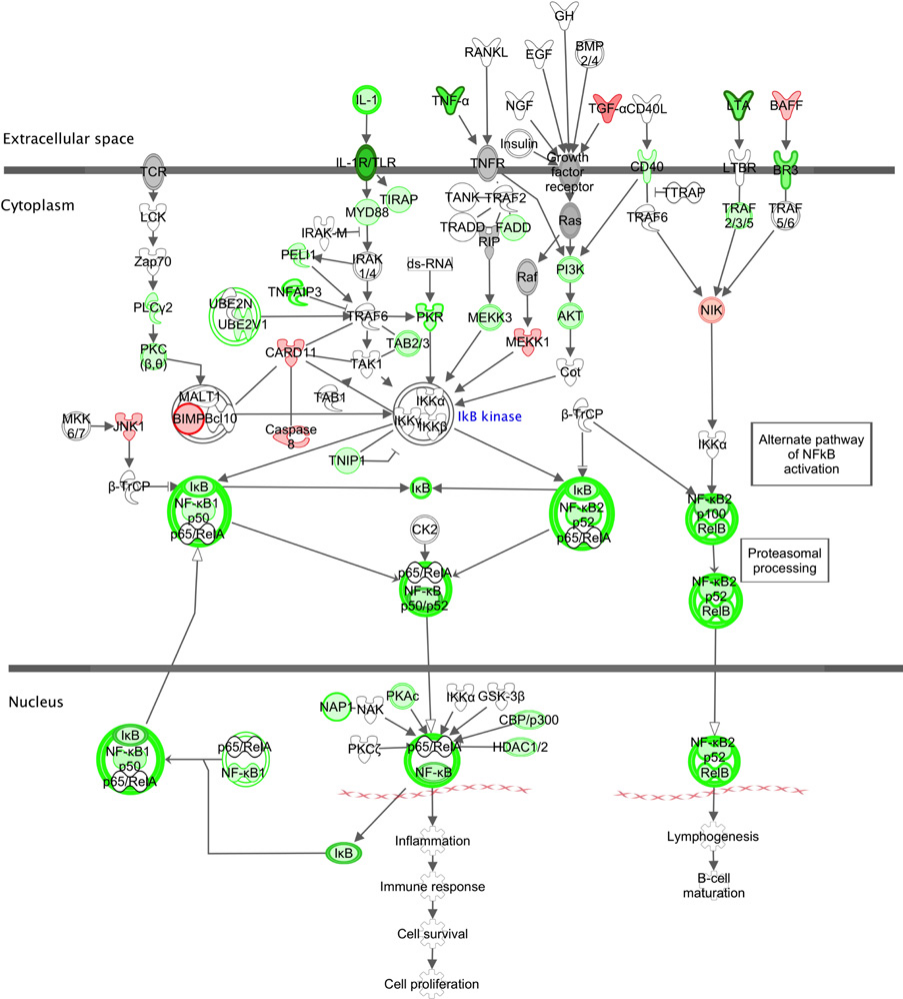
Canonical pathway for transcriptional activation by NFκB Explanation of symbols as in Fig 4.

Interleukin-1 (IL-1) signaling is flagged by IPA^®^ as a top down-regulated pathway (Table 4) with reduced expression of important pro-inflammatory members of the IL-1 superfamily [59]. For example, the mRNAs for IL-1β, its receptor IL-1R1 and accessory protein IL-1RAP are decreased 1.5-, 11.7- and 1.2-fold respectively while IL-18, IL-18R1 and IL-18RAP are down 2.3-, 3.0- and 4.0-fold respectively. Expression of pro-inflammatory *IL32* is also reduced 5-fold, while the expression of Tumor Necrosis Factor (*TNF* or *TNFα*), which can activate both Type I IFNs and the inflammatory mediator NF-κB, is down-regulated 30-fold (S3 Table). The canonical pathway leading to transcriptional activation by NF-κB through IL-1, TNFα and other ligands is shown in Fig 6. The NF-κB complex is an important mediator of inflammatory and immune responses and responds to PRRs and pro-inflammatory cytokines [60]. It can synergize with STAT signaling with the increased induction of target genes resulting from coordinate binding of STATs and NF-κB to GAS and NF-κB promoters. The p50 and p52 components of the NF-κB complex and the *RELB* transactivator are all down-regulated as are many components of signaling pathways that lead to NF-κB activation.

It is known that Type I IFNs and TNF can mutually suppress each other’s expression, and it has been suggested that changes in the cross-regulation of these pathways might affect the balance between the potential destructive and protective roles of these cytokines in the pathogenesis of autoimmune inflammatory diseases such as systemic lupus erythematosus (SLE) and rheumatoid arthritis (RA) [61]. IPA^®^ identifies signaling in RA as a top-ranked affected canonical pathway (Table 4) and the list of DEGs associated with RA and SLE are shown in S6 Table. Although the modest changes in expression in some other cytokine receptors could potentially be pro-inflammatory (e.g. *IL10RA* and *IL23R*), the overall picture is one of the suppression of inflammation by elevated Ap_4_A, with the down-regulation of NF-κB signaling featuring strongly.

#### Up-regulated canonical pathways and MHC class II antigens

KBM-7 cells can be regarded as immature precursors to professional antigen-presenting cells such as macrophages and dendritic cells and almost all of the top 20 up-regulated canonical pathways flagged by IPA^®^ involve functions associated with the adaptive immune response, including antigen presentation, OX40 signaling, allograft rejection and B cell development (Table 4 and S4 Table). However, it should be emphasized that this is largely because these pathways all involve one of the most prominent up-regulated gene sets, the inducible MHC class II antigens (MHC-II). MHC-II molecules are mainly concerned with the presentation of antigens derived from extracellular pathogens resulting in CD4+ T helper cell priming and the production of antibodies by B cells [62]. Almost all class II subtype genes show a significant increase in expression, with some showing a large increase, e.g. *HLA-DOA* 47-fold and *HLA-DPA1* 12-fold. Thus, the extent to which these canonical pathways can be regarded as up-regulated as a whole is open to question.

Nevertheless, in addition to MHC-II, a number of other genes involved in promotion of aspects of the adaptive immune response are up-regulated in NuKO cells. OX40 (CD134, TNFRSF4) is a member of the TNF receptor superfamily expressed by activated T helper cells and other cells and when engaged by the OX40L ligand promotes the clonal expansion of effector and memory T cells responding to an antigen [63]. It is up-regulated 5-fold. CD86 (up 2.2-fold) is expressed on antigen-presenting cells and interacts with T cell surface ligands. It is crucial for effective T cell activation and survival [64] and several studies have shown that down-regulation or blockade of CD86 can improve allograft survival [65]. FCER1G (up 8-fold) encodes the γ chain (FcRγ) of the high affinity IgE receptor FCεR1 [66]. FcRγ is also a component of several other Fc receptors and the T-cell receptor, which may explain its association by IPA^®^ with several immune functions. Interestingly, activation of mast cells by aggregation of FCεR1 with IgE-antigen complexes has been reported to promote synthesis of Ap_4_A by lysyl-tRNA synthetase and the consequent transcriptional changes [10, 31, 36]. Up-regulation of FCER1G might therefore provide a mechanism to potentiate and prolong Ap_4_A synthesis.

Together, these data suggest reinforcement of elements of the adaptive immune response by elevated Ap_4_A. As far as KBM-7 cells are concerned, this may indicate that Ap_4_A is promoting their differentiation from a blast-like phenotype to a more mature stage [67]. There are some apparent exceptions, such as the down-regulation of the recombination activating genes RAG1 and RAG2 (down 4.4- and 107-fold respectively). The RAG proteins catalyze VDJ recombination and are essential for the generation of mature, functional T and B cells [68]. These genes are moderately expressed in KBM-7 cells and can be highly expressed in other blast-phase CML-derived cell lines, e.g. NALM-1 [69]. However, their relevance to immune function is in lymphocytes, in which their expression is normally confined, and in that context they may not be down-regulated by high Ap_4_A.

#### Tryptophan catabolism

**Fig 7:**
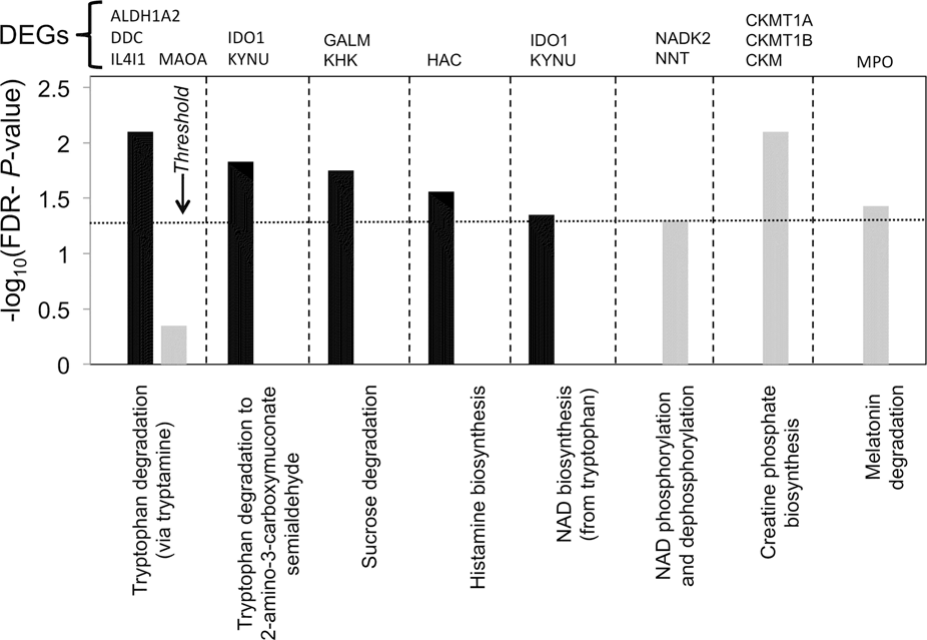
Top metabolic canonical pathways associated with down-regulated (black) and up-regulated (grey) gene sets. The dotted line represents the threshold above which there are statistically significantly more genes in a pathway than expected by chance (-log(FDR-adjusted *P*-value) >1.3).

Canonical pathway analysis also shows that a number of metabolic pathways including tryptophan (Trp) catabolism, and consequently *de novo* NAD+ biosynthesis (derived from Trp [70]), are strongly associated with the set of down-regulated genes (Fig 7 and S4 Table) while creatine phosphate biosynthesis, melatonin degradation (also a Trp derivative) and NAD+ phosphorylation are associated with the set of up-regulated genes. The strong down-regulation of both major pathways of Trp catabolism, particularly the key enzymes kynureninase (*KYNU*, 65-fold), indoleamine 2,3-dioxygenase (*IDO1*, 19-fold) and *DOPA* decarboxylase (Trp decarboxylase, *DDC*, 16-fold) is of particular note. Expression of the rate-limiting IDO1 is induced in myeloid-lineage cells by IFNs, particularly Type II, and TNF can act synergistically to increase *IDO1* expression [71], so the observed down-regulation of these pathways in NuKO cells combined with the moderate up-regulation of the negative effectors *DAP12* (*TYROBP*) and *BIN1*, a tumor suppressor, (S3 Table) [72] would be expected to reduce *IDO1* expression substantially.

There is strong evidence that Trp catabolism and *IDO1* expression in dendritic cells, which have a myeloid lineage, are important for the suppression of T-cell responses and the promotion of immune tolerance [73]. The reduction of extracellular Trp, the generation of metabolites via the kynurenine pathway and the signaling function of tyrosine-phosphorylated IDO1 all contribute to immunosuppression and protection against autoimmune disease and allograft rejection by inhibiting the proliferation of T cells and NK cells and promoting autophagy and anergy [74, 75]. The strong down-regulation of Trp catabolism caused by elevated Ap_4_A would therefore be expected to promote T-cell responses and suppress tolerance. This is consistent with the up-regulation of adaptive immune functions in NuKO cells predicted by IPA^®^ (Table 4). Furthermore, overexpression of *IMPACT*, an inhibitor of the GCN2-kinase (*EIF2AK4*) stress-signaling pathway that represses translation and proliferation in response to amino acid starvation, protects T-cells from Trp depletion [76]. *IMPACT* is up-regulated 13-fold in NuKO cells (S3 Table) and this would contribute further to the promotion of T-cell responses if reproduced in T cells.

#### Cancer

Introduction of Ap_4_A by cold shock into some cell lines has been reported to induce cell cycle arrest and apoptosis [19, 77]. It has also been shown that the level of *NUDT2* expression positively correlates with lower survival and increased lymph node metastases in breast carcinoma [78]. This suggests that low Ap_4_A might promote and/or high Ap_4_A might inhibit cancer progression. Given the multifactorial nature of the disease, it is not surprising that IPA^®^ classifies 1,108 of the 1,685 DEGs with ≥ 2-fold change and *P* ≤ 0.05 as being increased, decreased or affected (usually by mutation association) in cancer. So, to investigate further whether the level of Ap_4_A might correlate with cancer cell proliferation, survival or metastasis, we have further filtered this DEG set to exclude those with FPKM values < 0.3 for both WT and KO samples to focus attention on the more abundant transcripts and avoid any uncertainties about the biological impact of DEGs with low level expression. We then conducted a literature search on the 40 most strongly up-regulated and down-regulated annotated protein-coding genes satisfying these conditions (S7 Table). Existing experimental evidence suggests that reduced expression of 14 of the top 40 down-regulated genes would lead to a significant anti-cancer effect while reduced expression of only 2 might have a promotional effect. A further 3 could be pro- or anti-cancer depending on cellular context. Of the top 40 up-regulated genes, increased expression of 8 restricts cancer progression in other systems, while only 2 promote it and one could possibly do either (S7 Table). Thus, increased intracellular Ap_4_A seems to be associated overall with a strong anti-cancer effect. A more detailed appraisal of these genes follows. It is worth noting that the well-characterized FHIT tumor suppressor protein that binds both Ap_3_A and Ap_4_A [8, 79] is not expressed in KBM-7 cells, probably as a result of gene deletion [38].

Several genes showing a high degree of differential expression have been associated in other cell systems with the epithelial-mesenchymal transition (EMT)—the loss of cell-cell adhesion that initiates metastasis—and the reverse process, mesenchymal-epithelial transition (MET), which stabilizes and integrates the cancer cells into tissues:

1. *GFRA1* encodes a receptor for glial cell line-derived neurotrophic factor (GDNF) and is classified by edgeR as the most strongly down-regulated gene in the dataset (3221-fold). The proliferation of prostate cancer cells and their resistance to genotoxic treatment correlate directly with the level of *GFRA1* expression [80]. It is also up-regulated in breast carcinoma [81] while GFRA1 released by cells can promote cancer cell migration and invasion [82].
2. Expression of the transcription factors *OVOL1* and *OVOL2* in mesenchymal prostate cancer and poorly differentiated breast cancer cells induces MET and so inhibits their metastatic potential [83]. It has been proposed that the EMT/MET balance is regulated by the ratio of *OVOL1/2* (promote MET) to *ZEB1/2* (promote EMT) expression. *OVOL1* is up-regulated 719-fold in NuKO cells (although *OVOL2* expression is decreased 8-fold) while *ZEB1* and *ZEB2* are slightly down 1.4- and 1.2-fold respectively.
3. The secreted glycoprotein *EDIL3* has recently been identified as a novel inducer of EMT in hepatocellular carcinoma. It promotes cell migration, invasion and angiogenesis [84]. It is also up-regulated in oral squamous cell carcinoma [85] but is down-regulated 590-fold in NuKO cells.
4. The mRNA binding protein *IGF2BP1* promotes EMT while its knockdown reduces cell migration in various mesenchymal-like tumor cells [86]. It is down 122-fold.
5. *GJA-1* was recently characterized as a key gene for cervical cancer invasion and metastasis [87] and is down 120-fold in NuKO cells.
6. The transmembrane protein *TM4SF1* is overexpressed in many cancers and in the tumor vascular endothelium [88] with its level correlating with poor prognosis in glioblastoma [89]. Its down-regulation by an endogenous miRNA in prostate cancer cells inhibited migration and invasion [90]. It is down 70-fold.
7. *BRINP3* overexpression in pituitary gonadotrope cells promotes proliferation, migration, and invasion [91]. It is down 136-fold.
8. *MPZL2* expression is significantly decreased in breast carcinoma cells growth-arrested by siRNA knockdown of the migration and invasion regulatory PACE4 proprotein convertase [92]. Its expression is reduced 260-fold in NuKO cells.
9. The RAC1-activating guanine nucleotide exchange factor *PREX2*, which is frequently mutated in cancer and which promotes migration and invasion of various neoplasias [93, 94] is down 44-fold.
10. High expression of the HSP40 family member *DNAJC12* has been found to correlate with colorectal tumor progression and invasion and with a poor response to neoadjuvant concurrent chemoradiotherapy [95]. It is down-regulated 41-fold in NuKO cells.
11. Down-regulation of the transcription factor *FOXD3* promotes an EMT phenotype in breast cancer cells, causing proliferation and invasion both *in vivo* and *in vitro* while overexpression inhibits this phenotype [96, 97]. Similar results have been found with other cancers [98, 99]. It is up-regulated 16-fold in NuKO cells.
12. Overexpression of *NKD2*, a negative regulator of Wnt signaling, in metastatic osteosarcoma and breast carcinoma significantly reduces tumor growth and metastasis *in vivo* and decreases cell proliferation, migration and invasion *in vitro*, while down-regulation has the opposite effect [100, 101]. It is up-regulated 19-fold.
13. The leukemia inhibitory factor receptor *LIFR* has been shown to act as a suppressor of metastasis in hepatocellular carcinoma [102]. Increased *LIFR* activity has also been correlated with a reduction in the pool of breast cancer stem cells [103]. It is up-regulated 11-fold.

Several other prominent DEGs are known to affect growth and apoptosis in other systems. The Wilms’ tumor transcriptional regulator WT1 can exhibit both oncogenic and tumor suppressor activities depending on its association with specific co-regulators [104, 105]. For example, the co-repressor BASP1 interacts with WT1 in a complex with PHB and BRG1 to favor growth arrest and the induction of apoptosis over proliferation [106]. All these genes are well expressed in KBM-7 cells, with *BASP1* exhibiting 35-fold up-regulation in NuKO cells. Up-regulation of IFI44L is associated with melanoma and prostate cancer [107, 108] while overexpression of NKX2-2 is associated with Ewing’s sarcoma and fibromatosis [109]. They are down 224- and 125-fold respectively in NuKO cells. The homeobox transcription factor NKX3-1 is a prostate tumor suppressor [110] and its expression is increased 13-fold in NuKO cells. Overexpression of the coiled coil domain protein CCDC68 decreased proliferation and tumorigenicity of pancreatic ductal adenocarcinoma cells while allelic loss was found in about half the tumors examined [111]. It has also been identified as a possible tumor suppressor in colorectal cancer [112] and is up-regulated 11-fold in NuKO cells. Even genes with a more modest change in expression could have a profound anti-cancer effect; for example, Interferon Regulatory Factor 4 (IRF4), an important NF-κB-activated regulator of immune system development and the innate immune response [113], also plays an essential role in many lymphoid malignancies, and knockdown of its expression by only 50% is lethal to multiple myeloma cells [114, 115]. It is down-regulated 7-fold in NuKO cells.

Mixed results have been reported for a few of these DEGs in other contexts. For example, high expression of the cysteine-rich secretory protein CRISP3 (down 130-fold in NuKO cells) has been found in certain subtypes of prostate cancer [116] but down-regulation has been associated with oral squamous cell carcinoma [117]. A similar pattern has been found for the JCHAIN component of IgA and IgM (down 75-fold), which is up-regulated in prostate [108] but down-regulated in colorectal cancer [118]. The chemokine CXCL10 is another factor with both tumor-promoting and anti-tumor effects, the latter largely through its immunogenic action. It is overexpressed up to 40-fold in most types of cancer and can promote tumor cell growth and metastasis [119] but is down 66-fold in NuKO cells. Mixed results have also been reported for the Ig superfamily protein CD200 and the transmembrane co-receptor syndecan-4 (SDC4), with both pro- and anti-cancer roles suggested in different situations [120–122]. They are down 51- and up 35-fold in NuKO cells respectively.

The change in expression of a small number of genes in NuKO cells could potentially promote cancer. For example, reduced expression of early B-cell factor 1 (EBF1) has been found in Hodgkin lymphoma and appears to contribute to the loss of B-cell phenotype and consequent malignancy [123]. There is also evidence for a tumor suppressor role in mouse leukemia [124]. It is down-regulated 38-fold. Expression of the TNF family member CD70 is normally restricted to activated T and B-cells but it is activated in a wide variety of tumors where it promotes tumor cell expansion and survival [125]. It is up-regulated 17-fold. Finally, type II transglutaminase (TGM2) is up-regulated 10-fold in NuKO cells and may have an important role in maintaining survival, invasion and the metastatic behavior of a variety of tumors and cancer stem cells [126] while sphingosine-1-phosphate phosphatase 2 (SGPP2, up 63-fold) may be a target for the tumor suppressor miRNA-31 [127].

If the above set of prominent DEGs were expressed and responded in the same way to increased Ap_4_A in carcinomas, the overwhelming net effect could be the strong suppression of tumor growth and, particularly, metastasis. Furthermore, as indicated previously, IDO1 and several other enzymes of Trp catabolism are strongly down-regulated in NuKO cells. Increased *IDO1* expression is a characteristic of many cancer cells and assists them in avoiding clearance by the immune system, with the level of expression often correlating with poor prognosis [72, 128] and so inhibitors of IDO1 have been considered as novel immunotherapeutic adjuvants to conventional anti-cancer drugs [129, 130].

**Fig 8:**
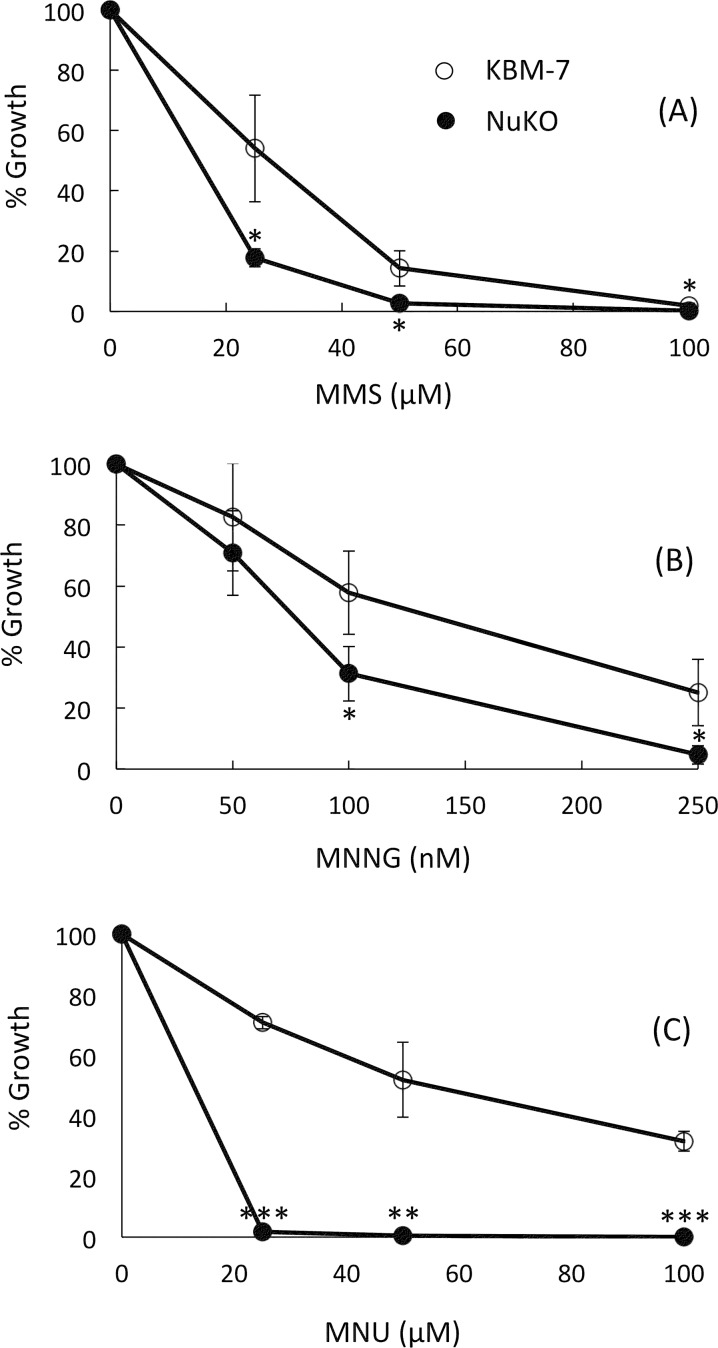
**Growth inhibition curves for cells in the presence of (A) methylmethane sulfonate (MMS), (B) *N*-methyl-*N*'-nitro-*N*-nitrosoguanidine (MNNG) and (C) *N*-methylnitrosourea (MNU).** Growth of KBM-7 (open symbols) and KBM-7-NuKO (closed symbols) cells was assessed as described in Materials and Methods. Data are presented as means ± SEM of the results obtained from three independent experiments. When no error bar is shown, the error is smaller than the symbol. Statistical significance was assessed by Student’s two-tailed t-test; *P < 0.05, **P < 0.01, ***P < 0.001 versus WT control.

Taken together, there seems to be sufficient evidence to support NUDT2 as a novel chemotherapeutic target that could conceivably exert an anti-cancer effect via multiple pathways involving apoptosis, metastasis, invasion and immunosuppression. One additional benefit of targeting NUDT2 could be the 158-fold down-regulation of O^6^-methylguanine-DNA methyltransferase (*MGMT*). The MGMT protein dealkylates toxic and mutagenic O^6^-alkylguanine lesions in DNA exposed to alkylating agents [131] and its reduced expression in NuKO cells renders them much more sensitive to growth inhibition by methylmethane sulfonate (MMS), *N*-methyl-*N*'-nitro-*N*-nitrosoguanidine (MNNG) and *N*-methylnitrosourea (MNU) (Fig 8). While down-regulation by high Ap_4_A would in theory promote the carcinogenic effect of environmental alkylating agents, it would also render cancer cells more sensitive to alkylation therapies such as temozolomide, a common treatment for glioblastoma and astrocytoma. MGMT status is an important determinant of the success of these therapies [132]. This sensitivity to methylating agents also provides good phenotypic confirmation of the transcriptomic data.

#### IPA^®^ prediction of upstream regulatory factors

**Fig 9:**
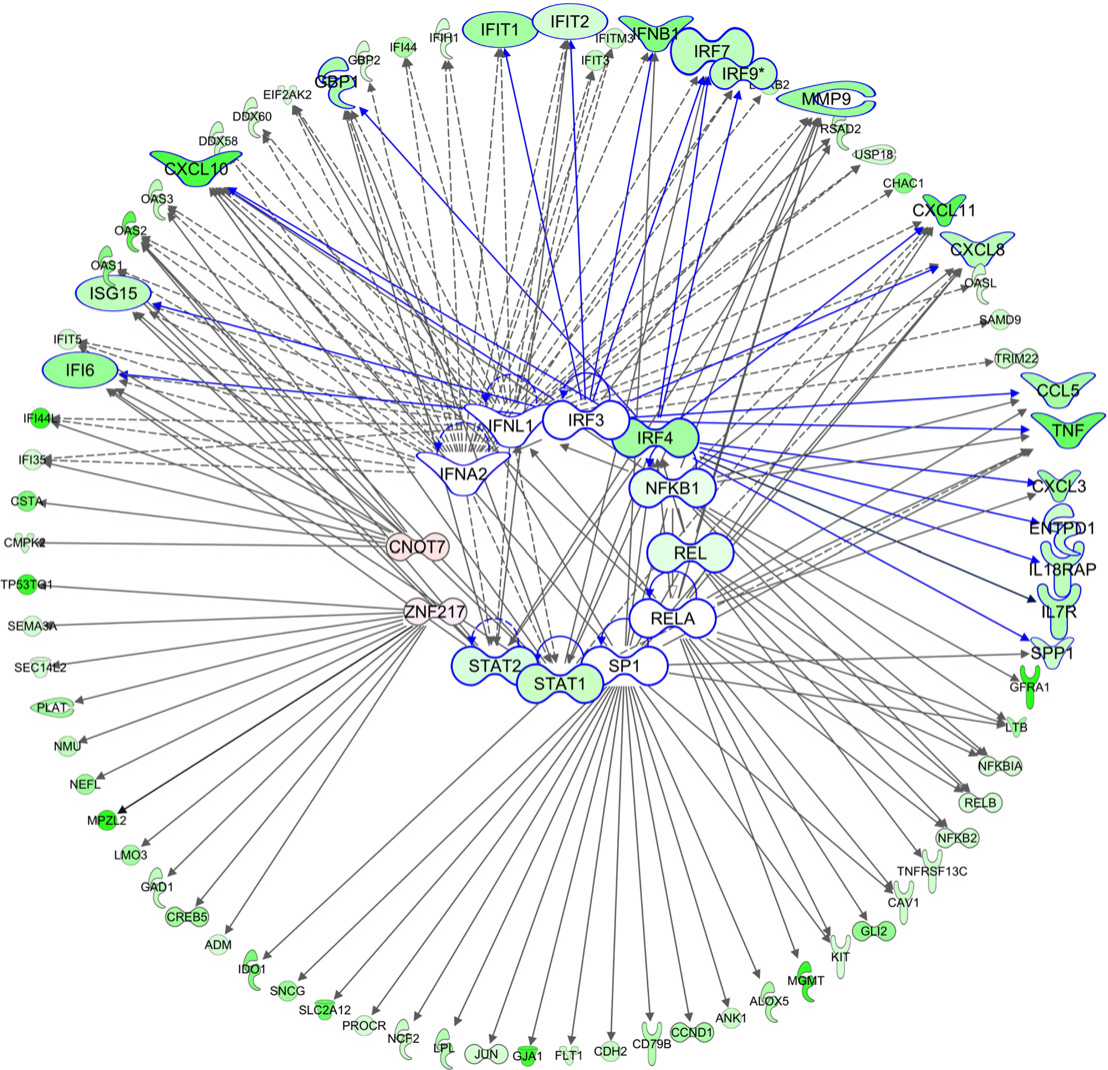
The top-ranked upstream transcription factors (in centre) suggested by IPA^®^ for the set of down-regulated genes (outer circle). The top two-ranked cytokines, IFNA2 and IFNL1 are also included. Genes with >1.2 fold down-regulation are in green; those with >1.2-fold up-regulation are in red. Color intensity corresponds to the fold change. Genes corresponding to immediate downstream targets of IRFs are enlarged with blue borders and connections.

IPA^®^ prediction of key upstream regulators for the DEGs suggests numerous factors controlling large sets of down-regulated genes with fewer factors controlling small groups of up-regulated genes. Of the top 100 most significant potential regulators, 97 are proposed to contribute to gene down-regulation (S8 Table) although the total number of genes that are up- and down-regulated is similar. The inter-relationships between the top-ranked transcription factors implicated in gene down-regulation and their major identified targets are shown in Fig 9. The functions of several of these have already been described. In addition, CNOT7 (hCAF1) is a STAT1-binding negative regulator of Type I and Type II IFN signalling [133], while the transcription factor IRF3 is an important responder to PRR activation (Fig 5) and co-operates with NF-κB and IRF7 in the transcription of IRF3- and NF-κB-dependent genes [113]. SP1 controls the transcription of multiple genes, many of which have been described as promoting the ‘hallmarks’ of cancer: proliferation, independence from growth signals, avoidance of apoptosis and immune destruction, invasion and metastasis, and angiogenesis. It is overexpressed in many tumors, making it a target for chemotherapy [134, 135].

Control of up-regulated genes appears to be less integrated with different factors potentially regulating small subsets of genes (S8 Table). Furthermore, of the top five listed regulators (*SMC3*, *PDLIM2*, *EBI3*, *MYOC* and *NEUROG1*, Table 4), little or no expression of *EBI3*, *MYOC* and *NEUROG1* was detected so their significance is hard to determine. In the case of MHC-II genes, SMC3, a subunit of the cohesin protein complex, was identified as a possible regulator. Cohesin co-operates with MHC-II specific transcription factors RFX5 and CIITA (up 1.3-fold) to activate MHC-II expression [136]. SMC3 has an ATP-binding domain but whether cohesin binding to MHC-II transcriptional insulators could be promoted by Ap_4_A is unknown. The ubiquitin E3 ligase PDLIM2 (SLIM) is predicted by IPA^®^ to both up-regulate and down-regulate different sets of genes (S8 Table). This prediction appears to be based largely on the results of one previous investigation into PDLIM2-modulated gene expression in DU145 prostate carcinoma cells [137] and the direction of expression change of about half the genes in that study is different from that in ours. Given that the *z*-scores for the predictions are both below the significance threshold of 2<*z*<-2, the reliability of this prediction is perhaps questionable. Nevertheless, *PDLIM2* suppression is known to alter the stability of several of the transcription factor families implicated here in the down-regulated responses in NuKO cells including NFκB, IRFs, STATs, JUN and AP-1, and also to promote reversal of the EMT phenotype [137]. Although the level of *PDLIM2* expression in KBM-7 cells was not significantly affected by *NUDT2* knockout (S2 Table), its activity may have been altered as a secondary effect of some other change e.g. to a protein kinase. Overall, though, there is no clear upstream pattern of regulation for the sets of up-regulated genes.

### Possible direct targets for Ap_4_A-mediated gene regulation

While IPA^®^ may have identified potential upstream regulatory factors for some of the observed changes in gene expression, the question still remains as to how the elevated level of Ap_4_A impacts on these and other factors to promote these changes. A number of possibilities can be considered.

#### HINT1

This study was initiated by the suggestion of Razin and co-workers that direct activation of MITF and USF2 transcription complexes in mast-related cells occurred by Ap_4_A-mediated displacement of the HINT1 co-repressor. For example, mast cell protease rMCP-6 (*TPSB2*), c-Kit (*KIT*), granzyme B (*GZMB*) and tryptophan hydroxylase (*TPH1*) were all up-regulated in RBL cells either after direct introduction of Ap_4_A, after activation by IgE and antigen, or after increasing Ap_4_A by siRNA knockdown of *NUDT2* [10, 31]. However, IPA^®^ did not identify MITF (up 1.7-fold), USF2 or HINT1 (down 1.3-fold) as potential regulators in our study. Furthermore, *TPSB2* was up 3.2-fold but *KIT* was down 2.3-fold and *TPH1* unaffected (S2 and S3 Tables). No data were retrieved for *GZMB*. The USF2-responsive genes *TGFB2*, *SHP* (*NR0B2*), *TERT* and *TSP-1* (*THBS1*) were also shown to be up-regulated by Ap_4_A in RBL cells [36]. In our study, *USF2* expression was unchanged by *NUDT2* knockout as was the very low-level expression of *TGFB2*, *TERT* and *THBS* (S2 Table). No data were obtained for *NR0B2*. Elevated Ap_4_A was also found to activate the MITF-dependent transcription of myosin light chain 1a (*MYL4*) in cardiomyocytes [10], but this gene is not expressed in KBM-7 cells. From a list of 113 genes shown to be up-regulated by MITF in melanoma [138], 23 were up- and 20 down-regulated in NuKO cells, which would suggest that Ap_4_A-mediated MITF or USF2 activation is not prominent in our data.

Other genes repressed by HINT1 through its interaction with MITF, USF2 or the WNT/β-catenin complex include cyclin D1 (*CCND1*), *BCL2*, *BIRC5*, *MET*, *MYC*, *FRA1*, *TGFB*, *HIF1A*, *PAI-1* (*SERPINE1*) and *AXIN2* [139–141]. Of these, *BCL2*, *MYC* and *AXIN2* were slightly up-regulated 1.3-, 1.3- and 2.4-fold while *CCND1* was down-regulated 6.2-fold. Loss of HINT1 has also been reported to reduce the expression of p21^WAF1/CIP1^ (*CDKN1A*), *GADD45A*, *GADD153* and *TP53INP1* [142] but only the predicted slight reduction in *CDKN1A* expression (1.4-fold) was observed in NuKO cells. Thus, there is no clear evidence to support the relief of HINT1 repression of transcription factors as a cause of Ap_4_A-mediated differential gene expression in KBM-7 cells.

#### Purinoceptors

Some Ap_4_A-mediated transcriptional changes could also involve externalization of Ap_4_A into the growth medium by exocytosis or from damaged or necrotic cells. This Ap_4_A could then act through cell-surface purinoceptors to modulate known signaling pathways leading to changes in gene expression [143]. All known P2 receptor subtypes except *P2X2*, *P2X3*, *P2X6*, *P2Y4*, *P2Y12* and *P2Y13* were found to be expressed in KBM-7 cells with *P2X1*, *P2X7* and *P2Y8* showing slight but significant up-regulation (1.3–2.3-fold) in NuKO cells (S2 and S3 Tables). Several of these are known to be activated by Ap_4_A [144]; however, measurement of Ap_4_A in the cell-free growth medium showed that there was actually less in the medium taken from NuKO cells (0.11 ± 0.01 pmol/10^6^ cells, *n* = 3) than in that from KBM-7 cells (0.28 ± 0.07 pmol/10^6^ cells, *n* = 3), possibly due to the 5.5-fold up-regulation of the cell surface phosphodiesterase ENPP1, which is known to hydrolyze extracellular diadenosine polyphosphates [3]. Thus, an increased autocrine signaling effect of Ap_4_A seems unlikely.

#### Chromatin remodeling

PARP1 and PARP2 are known to be intimately involved in chromatin (de)condensation and epigenetic marking through ADP-ribosylation of histones and chromatin remodeling enzymes and via interaction with numerous transcription factors, and this has profound effects on gene expression [145]. Ap_4_A can effectively compete with histones as an ADP-ribose acceptor resulting in the synthesis of ADPR-Ap_4_A species [17, 146, 147] and so an elevated level of Ap_4_A could conceivably regulate, or just interfere with, these processes leading to changes in gene regulation. However, the lack of effect on the expression of the selected gene set studied by qRT-PCR after inhibition of PARP1 and PARP2 would argue against a major role for PARPs. Nevertheless, the expression of a large number of histone gene variants is affected by *NUDT2* disruption, the majority being down-regulated, while changes in the expression of several lysine-specific demethylases (KDMs), histone deactylases (HDACs) and DNA (cytosine-5)-methyltransferases (DNMTs), several of which are also ADP-ribosylation targets, is also evident (S3 Table). Hence, Ap_4_A-mediated chromatin remodelling by some unknown mechanism could still be considered as a potential source of differential gene expression.

#### Protein kinases and other ATP-dependent factors

By virtue of its structural similarity to ATP, Ap_4_A might regulate transcription by inhibition of protein kinases, of which there are over 500 in the human genome [148]. Ap_4_A has been shown to inhibit v-Src [26], casein kinase II [27] and protein kinase C [28]. These and many other protein kinases, some of which might be particularly sensitive to Ap_4_A, are known to regulate transcription factor activity directly or indirectly and several examples of potential targets feature in Figs 4–6. In the absence of evidence strongly favoring alternative possibilities, protein kinases and other ATP-dependent regulatory factors such as phosphoinositide kinases, chaperones and ABC transporters must be regarded as likely targets for Ap_4_A that will require future investigation.

#### Transcript stability

An alternative mechanism whereby increased Ap_4_A could have a major effect on the transcriptome is through inhibition of RNA binding by the nudix protein NUDT21 (CFIm25, CPSF5), the 25 kDa component of the cleavage factor Im complex involved in pre-mRNA 3′-end processing. Ap_4_A binds to the same site as RNA with a *K*_d_ of 2.4 μM and so might be expected to affect 3′-end processing and the half-lives of certain mRNAs [35, 149]. Knockdown of *NUDT21* in glioblastoma cells results in shortened 3′-UTRs in 1450 transcripts and an increase in cell proliferation [150]. Of these 1450 transcripts, the steady-state levels of 928 were significantly increased and 28 were decreased. However, a comparison between the transcripts affected after *NUDT21* knockdown in glioblastoma cells and those up- and down-regulated in NuKO cells revealed no overlap between the down-regulated genes and only 1 overlap in the top 250 up-regulated genes. Therefore, despite the differences in cell lines, it seems unlikely that NUDT21 is an important target for the increased Ap_4_A in NuKO cells.

#### Alternative substrates or effects of NUDT2 disruption

Other *in vitro* substrates for NUDT2 whose levels might be affected by its loss include Ap_5_A, Ap_6_A, other homodinucleoside polyphosphates with four or more phosphoryl groups, e.g. diguanosine tetraphosphate (Gp_4_G) [4, 5], inorganic polyphosphate [151] and phosphoribosyl pyrophosphate [152], but there is no evidence to suggest that these are significant substrates *in vivo* [4]. Ap_5_A and Ap_6_A appear to be confined to secretory granules in certain specialized cells while no specific mechanisms are known for the synthesis of Gp_4_G and other homodinucleoside polyphosphates in mammalian cells [4, 5]. Heterodinucleoside polyphosphates such as Ap_4_G and Ap_4_U can also be synthesized by aminoacyl-tRNA synthetases and, if present, would be detected by our luminometric assay and included as part of the ‘Ap_4_A’ pool, but as no unique functions have been ascribed to these molecules they are usually considered under the heading ‘Ap_4_A’ [4].

Transcript stability could also be affected if NUDT2 were involved in mRNA decapping. So far, two related nudix family proteins, DCP2 (NUDT20) and NUDT16, have been shown to participate in mRNA decapping *in vivo* [153]. However, a further six nudix proteins, including NUDT2, have varying degrees of decapping activity *in vitro* on both monomethylated and unmethylated capped RNAs [154]. Although there is currently no evidence supporting NUDT2-mediated decapping *in vivo*, this possibility cannot be discounted. Additionally, by virtue of its structural similarity to caps, elevated Ap_4_A could conceivably inhibit decapping by DCP2 and NUDT16. In both cases, this would most likely lead to prolonged half-lives of mRNA subsets and might therefore contribute to the up-regulation of certain genes.

Finally, loss of NUDT2 could have consequences through the loss of interaction with any binding partner. NUDT2 may have a significant nuclear location [155, 156] and has been documented to bind to the replicative helicase component MCM6 [157]. While this may in some way be related to the inhibition of replication initiation by Ap_4_A [17], it is not clear how loss of this interaction would have the profound effect on transcription observed in NuKO cells. NUDT2 has also been reported to bind to unliganded estrogen receptor beta (ESR2) in the cytosol [158]. This is interesting given the reported repression of NUDT2 expression by estradiol [78, 159]. However, no significant expression of *ESR2* was detected in KBM-7 cells (S2 Table and [38]) and so it seems unlikely that the effects of NUDT2 disruption involve ESR2-mediated gene expression. Thus, aside from a theoretical effect of NUDT2 loss on mRNA decapping, it seems reasonable to conclude that most of the transcriptional effects reported here are caused by an increased level of its major substrate, Ap_4_A. We did attempt to answer this question directly by expression of the *Escherichia coli ApaH* gene in NuKO cells. *ApaH* encodes a symmetrically-cleaving Ap_4_A hydrolase that it structurally unrelated to NUDT2 [160] and in so doing we hoped to reduce Ap_4_A to normal levels in a NUDT2-negative background. However, *ApaH* expression proved to be toxic to the cells, possibly because ApaH may also have protein phosphatase activity [161].

## Discussion

Despite being known since the 1960s, Ap_4_A has never commanded the attention that has been bestowed on other low-molecular-weight regulators such as cyclic nucleotides and inositol phosphates. Two competing schools of thought have arisen, one suggesting that Ap_4_A is a physiologically important regulator whose level is finely tuned by the NUDT2 Ap_4_A hydrolase, and the other that it is an unavoidable, non-functional by-product of several enzyme activities and that NUDT2 exists simply to eliminate it, lest it cause molecular mayhem by interfering with essential, adenine nucleotide-dependent metabolic and regulatory pathways [4]. The data presented here clearly demonstrate that increases in intracellular Ap_4_A by disruption of a single gene lead to significant changes to the transcriptional program. While some of the observed changes in gene expression may indeed be adventitious due to an unregulated and sustained high level of Ap_4_A, the specific down-regulation of gene sets involved in the interferon, inflammatory and innate immune responses and in cancer promotion support the view that Ap_4_A is indeed a biologically relevant regulator. Assuming that Ap_4_A has more than one intracellular target, it is likely that different gene sets will respond to different levels of Ap_4_A resulting from the regulation of NUDT2 activity, translation or transcription *in vivo* in response to different factors, and so not all the effects observed in NuKO cells will necessarily occur at the same time. Identification of these targets and the gene networks under their control is a priority for future work.

Taking the positive view that Ap_4_A is a *bona fide* regulator, what conclusions can be drawn about its principal intracellular role(s)? Rapid suppression of interferon responses after activation of the initial signal transduction pathways is an essential part of the overall immune response to pathogens to avoid the potential toxicity of the many anti-viral, pro-apoptotic, and anti-proliferative proteins that are induced. Therefore, the NuKO phenotype may reflect the activation of these feedback mechanisms. Alternatively, components from infecting pathogens such as proteins or 5′-ppp RNAs may actively cause the increased intracellular Ap_4_A by inhibiting NUDT2 in order to down-regulate the immune responses. Poliovirus infection is known to cause a slight (2-fold) increase in Ap_4_A [162] while the SARS coronavirus protein 7a physically interacts with NUDT2, although the effect of this on the level of Ap_4_A is not known [163]. Recently, it has been shown that the viral-induced mediator of the interferon response, cyclic GAMP, can be transferred from cell to cell inside newly-formed virions, and it has been speculated that this is a protective, host-regulated mechanism to rapidly establish an antiviral state in newly infected cells [164, 165]. It is equally interesting to speculate that viruses may also package Ap_4_A into new virions to counteract this. With regard to bacterial pathogens, the two types of bacterial Ap_4_A hydrolase, the asymmetrically-cleaving NUDT2 homologue RppH (also known as YgdP or IalA) and the unrelated symmetrically-cleaving ApaH, have been classified as invasion proteins and are required for optimal survival of bacteria during cellular invasion [166–169]. They may help to prevent high host cell Ap_4_A induced by infection-associated stress from inhibiting essential bacterial functions.

The down-regulation of tryptophan catabolism by Ap_4_A offers a possible explanation as to why, of all the aminoacyl-tRNA synthetases able to generate diadenosine oligophosphates, mammalian tryptophanyl-tRNA synthetase (WRS) is the only one unable to synthesise Ap_4_A. It can only make Ap_3_A [170]. WRS is expressed constitutively in all cells, but can be strongly induced in many non-lymphoid cells, e.g. monocytes, by Type II interferons, leading to a marked increase in Ap_3_A, but not Ap_4_A [171, 172]. It has been proposed that this induction protects non-lymphoid cells from Trp depletion and the other effects of IDO1 expression by ensuring that sufficient Trp is diverted into protein synthesis for survival [173]. Enhanced WRS expression in T cells from patients with several autoimmune disorders is also believed to protect them from Trp depletion [72]. If this increased level of WRS were also to generate a significant amount of Ap_4_A, this would compromise the intended immunosuppression by down-regulating Trp catabolism. Hence, WRS may have evolved a unique inability to make Ap_4_A. This also seems to confirm the physiological relevance of Ap_4_A as an important signaling molecule.

Regardless of the mechanisms where by Ap_4_A exerts its effects and whether these are all physiologically relevant, the practical significance of inhibiting NUDT2 is evident. Our results expand upon the earlier demonstration that NUDT2 promotes proliferation of breast carcinoma cells and that NUDT2 status could be a useful prognostic marker [78]. The potential of NUDT2 as a pleiotropic therapeutic target for cancer simultaneously affecting metastasis, invasion, apoptosis, immunosuppression and inflammation certainly warrants further investigation and validation in different cancer cells and animal models. There are currently no known specific small molecule inhibitors of NUDT2; however such molecules have been described for the related nudix hydrolase MTH1 [174, 175], suggesting that specific inhibition of NUDT2 may be feasible. It could also be targeted with biotherapeutics. The extent to which the changes in the expression of other genes and pathways brought about by *NUDT2* disruption might militate against its value as a target will only be determined by further investigation.

## Supporting Information

S1 Table Primers used for qRT-PCR verification of RNA-Seq data. (DOCX)

S2 Table Raw data for the 31,177 reads mapped to the human reference genome. Data include Ensembl gene id, chromosome mapping data, log_2_ counts per million mapped reads (CPM), log_2_ fold changes for four contrasts, *P*-values and FDR-adjusted *P*-values, raw counts and individual sample and mean FPKM values. The table does not contain the genes with zero read counts in all libraries.
(XLSX)

S3 Table List of the subset of 4,835 genes showing differential expression between KBM-7 and KBM-7-NuKO cells with fold-change ≥ 1.2 and *P* <0.05. Data include Ensembl and gene id, log_2_ CPM, mean FKPM value of triplicate KO and WT samples, log_2_ fold change, fold change (up-regulation in red, down-regulation in green), FDR-adjusted *P*-value and approved gene name. List includes unannotated genes, pseudogenes and some non-protein coding genes.
(XLSX)

S4 Table Mapping of DEGs to Ingenuity® canonical pathways. Data include the observed overall direction of regulation [down (-) or up(+)], -log_2_(*P*-value), *z*-score and key molecules in each pathway.
(XLSX)

S5 Table **DEGs from this study previously shown to be regulated by (A) Type I, (B) Type II and/or (C) Type III interferons according to the Interferome v2.01 database**. Data include Ensembl id, gene id and description, and fold up- (red) or down- (green) regulation in this study. Genes potentially showing specific regulation by Type III IFNs are highlighted in yellow.
(XLSX)

S6 Table Genes identified by IPA^®^ to be associated with rheumatoid arthritis and systemic lupus erythematosus. (XLSX)

S7 Table Top 40 down- and top 40 up-regulated genes with *P* ≤ 0.05 and FPKM >0.3 and their association with cancer. (XLSX)

S8 Table Upstream regulators predicted by IPA^®^. Data include the predicted upstream regulator, the direction of regulation with which it is associated (- down; + up), the type of molecule, the activation state (predicted direction of the biological function), the activation *z*-score, where >2.0 or <-2.0 is significantly predictive, the *P*-value and the molecules for which there is documented evidence for regulation. The top transcription factors examined in Fig 9 are highlighted in green.
(XLSX)
