# Supplementary material for: NUDT2 Disruption Elevates Diadenosine Tetraphosphate (Ap4A) and Down-Regulates Immune Response and Cancer Promotion Genes
Source: PLoS One. 2016 May 4;11(5):e0154674. doi: 10.1371/journal.pone.0154674 (PMC4856261; doi:10.1371/journal.pone.0154674)
Supplement: S1 Table — (DOCX) [file pone.0154674.s001.docx]

S1 Table. List of primers used for qRT-PCR

| Gene | Ensembl id |  | 5'-->3' |
| --- | --- | --- | --- |
| GAPDH | ENSG00000111640 | For | GTGGAAGGACTCATGACCA |
| (reference) |  | Rev | GAGGCAGGGATGATGTTCT |
| CXCL11 | ENSG00000169248 | For | CCTGGGGTAAAAGCAGTGAA |
|  |  | Rev | TGGGATTTAGGCATCGTTGT |
| OAS2 | ENSG00000111335 | For | GAGACACGTCCGAACTCACA |
|  |  | Rev | TACGCGTACTTCTGCTGCAC |
| TNF | ENSG00000232810 | For | AACCTCCTCTCTGCCATCAA |
|  |  | Rev | GGAAGACCCCTCCCAGATAG |
| KYNU | ENSG00000115919 | For | CCAAGAGAGGGGGAAGAAAC |
|  |  | Rev | GCTTTTGTGATGGCAGGAAT |
| SDC4 | ENSG00000124145 | For | TCGATCCGAGAGACTGAGGT |
|  |  | Rev | CCAGATCTCCAGAGCCAGAC |
| CAMK2D | ENSG00000145349 | For | CCTTCTGGGATGAAGACCAA |
|  |  | Rev | CGTTTGGCAGGGTTGATAGT |
| INHBA | ENSG00000122641 | For | GGAGGGCAGAAATGAATGAA |
|  |  | Rev | CCTTGGAAATCTCGAAGTGC |
| NKX2-1 | ENSG00000136352 | For | GGACGTGAGCAAGAACATGG |
|  |  | Rev | CGCCGACAGGTACTTCTGTT |
| OVOL1 | ENSG00000172818 | For | GAGACACGTCCGAACTCACA |
|  |  | Rev | TACGCGTACTTCTGCTGCAC |
| NFKB2 | ENSG00000077150 | For | TACCTGGTGATCGTGGAACA |
|  |  | Rev | GATAGGTCTTTCGGCCCTTC |
| JUN | ENSG00000177606 | For | TGACTGCAAAGATGGAAACG |
|  |  | Rev | CAGGGTCATGCTCTGTTTCA |
| IRF7 | ENSG00000185507 | For | TACCATCTACCTGGGCTTCG |
|  |  | Rev | TGCTGCTATCCAGGGAAGAC |
| IL18 | ENSG00000150782 | For | GAGAAGTGTCCCAGGACATGA |
|  |  | Rev | CCCCCAATTCATCCTCTTTT |
| IL1R1 | ENSG00000115594 | For | ATTGATGTTCGTCCCTGTCC |
|  |  | Rev | TGAATCCTGGAGGCTTGTTC |
| HLA-DPB1 | ENSG00000223865 | For | TTTCTACCCAGGCAGCATTC |
|  |  | Rev | TCACCAGGATCTGGAAGGTC |
| HLA-DPA1 | ENSG00000231389 | For | CGCCCTGAAGACAGAATGTT |
|  |  | Rev | CGGCATAAGTTGACACATGG |
| GFRA1 | ENSG00000151892 | For | TGGAGGATTCCCCATATGAA |
|  |  | Rev | TTCTTGCAAATGTCGTCGAG |
| BRINP3 | ENSG00000162670 | For | CGGAACCTGGGCTATATGAA |
|  |  | Rev | GCAAAAGTGCTGAATCCTG |
| MGMT | ENSG00000170430 | For | CCGTTTGCGACTTGGTACTT |
|  |  | Rev | CTCACAACCAGACAGCTCCA |
| BASP1 | ENSG00000176788 | For | GGCTACAATGTGAACGACGA |
|  |  | Rev | CTTCTCCTTGCCCTCCTTG |
